# Elongases of Long-Chain Fatty Acids ELO2 and ELO9 Are Involved in Cuticle Formation and Function in Fecundity in the Yellow Fever Mosquito, *Aedes aegypti*

**DOI:** 10.3390/insects14020189

**Published:** 2023-02-14

**Authors:** Jing Chen, Yu-Chen Wu, Jiu-Kai Chen, Xiao-Jing Zhu, David Merkler, Cheng-Hong Liao, Qian Han

**Affiliations:** 1 Laboratory of Tropical Veterinary Medicine and Vector Biology, School of Life Sciences, Hainan University, Haikou 570228, China; 2 One Health Institute, Hainan University, Haikou 570228, China; 3 Department of Chemistry, University of South Florida, Tampa, FL 33620, USA

**Keywords:** *Aedes aegypti*, elongase, cold tolerance, cuticle, development, fecundity

## Abstract

**Simple Summary:**

Elongases of long-chain fatty acids (ELOs) play an important role in the function and metabolism of fatty acids of different chain lengths. However, little is known about the expression and biological function of fatty acid ELOs in insects, including in the larvae and adult blood-feeding *Ae. aegypti* mosquitoes. Therefore, the study of ELOs is necessary to understand the development and reproduction of insects, including mosquitoes. In this study, two novel *ELO* genes were identified in *Ae. aegypti.* We used two different RNAi methods (larval-nanoparticle feeding; adult-microinjection) to analyze the functions of *AeELO2* and *AeELO9* in larvae development and adult fecundity in *Aedes aegypti*. The results indicated that *AeELO2* and *AeELO9* play crucial roles in larval development and the adult fecundity of *Ae. aegypti*. *AeELO2* mainly participated in larval molting behavior and growth, and it regulated the flexibility and elasticity of adult mosquito cuticles. *AeELO9* affected the cold resistance of larvae and adult mosquitoes. It also regulated the permeability of larvae and adult mosquito cuticles and egg development, therefore affecting the reproductive ability of *Ae. aegypti.*

**Abstract:**

Long-chain fatty acid elongases (ELOs) play important roles in the metabolism of fatty acids in insects. In this study, the genes for two elongases from *Aedes aegypti* were identified, *AeELO2* and *AeELO9*. Quantitative real time PCR showed that *AeELO2* and *AeELO9* are expressed at all developmental stages and some body parts, but with different expression patterns. RNAi-mediated knockdown of *AeELO2* and *AeELO9* was performed to investigate their roles in the development, growth, osmotic balance, and cold tolerance of *Ae. aegypti*. Knockdown of *AeELO2* slowed larval growth and development by causing molting abnormalities. Additionally, 33% ± 3.3% of adults died during oviposition, accompanied by an abnormal extension of cuticles in *AeELO2*-dsRNA knockdown mosquitos. Knockdown of *AeEL09* resulted in abnormal balance of cuticular osmotic pressure and a reduction in egg production. The maximal mRNAs of *AeELO2* and *AeELO9* were detected in eggs at 72 h after oviposition. Moreover, *AeELO2* knockdown reduced the egg hatching rates and *AeELO9* knockdown larvae did not develop well. In summary, *AeELO2* is involved in larval molting and growth, and its knockdown affects the flexibility and elasticity of adult mosquito cuticles. *AeELO9* regulates cold tolerance, osmotic balance, and egg development in *Ae. aegypti*.

## 1. Introduction

Fatty acids are the most important energy storage molecules in insects and act as the basic material for insect embryonic development, metamorphosis, flight, and other functions. In addition, they are vital components of insect cuticles, somatic membranes, and are the precursor of many insect sex pheromones. Some fatty acids are associated with the cold resistance of insects. 

**Figure 1:**
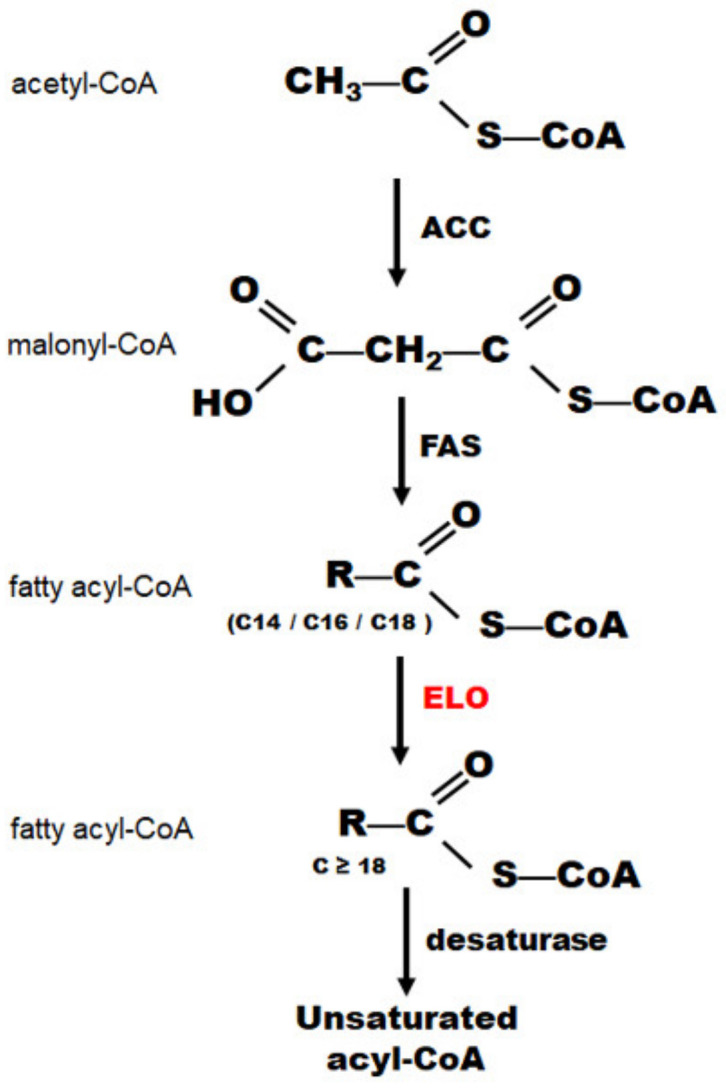
Overview of the fatty acid biosynthesis pathway. Additionally, the pathway comprises: (1) conversion of acetyl-CoA to malonyl-CoA by acetyl-CoA carboxylase (ACC); (2) conversion of malonyl-CoA to fatty acyl-CoAs by fatty acid synthase (FAS); (3) elongation of fatty acyl-CoAs by long-chain fatty acid elongases (ELOs); (4) desaturase conversion of saturated fatty acids into unsaturated fatty acids.

The long-chain fatty acid elongases (ELOs), the rate-limiting condensing enzymes in the first step of the elongation cycle (Figure 1), play important roles in the function and metabolism of fatty acids of different chain lengths (C18-C26) [1,2,3]. They are involved in various insect functions, including mating, reproduction, pheromone biosynthesis, and cuticle formation [4,5]. A few earlier studies have emphasized that the requirement of the elongases in growth, development, and survival is likely conserved in organisms. In *Drosophila melanogaster*, RNAi-mediated knockdown of a gene for a predicted elongase, *CG6660*, induced a lethal phenotype [6]. An essential gene, *neighbor of abl* (*noa*), encoding a very long-chain fatty acid elongase, has a somatic role in *Drosophila* development [7]. In *Tenebrio molitor*, RNAi-mediated silencing of *TmELO1* increased the mortality rate indicating that *TmELO1* is essential for mealworm survival [8]. Similar requirements for elongases in organism survival have been reported in other species [9,10]. An analysis of the expression pattern of the *ELO* gene in *Apis mellifera* revealed that ten *ELO* genes may be associated with cuticle hydrocarbon biosynthesis and have important roles in cuticle development [8]. In the brown planthopper, *Nilaparvata lugens*, nine *NlELO* genes were essential for the survival of nymphs and adults [11]. In addition, elongases are required for neonatal survival in mice [5], for the survival of the protozoan parasite, *Toxoplasma gondii* [10], and for the growth and survival of cancer cells [12]. 

In insects, the cuticle has dissimilar properties at different developmental stages and in different body parts. During early developmental stages, the cuticle becomes flexible and elastic, and becomes sclerotized and melanized shortly after molting [13,14,15,16]. This process occurs periodically during the growth and development of insects. The hard exoskeleton acts as a protective barrier, while the flexible cuticle allows the insects to move [13,17]. *Ae. aegypti* needs to molt continuously during the larvae stage. In previous studies, it was found that 3,4-dihydroxypheny-lacetaldehyde (DOPAL) synthase is directly linked to the flexible cuticle proteins (CPs), cross-linking them through the interaction of free amino acid groups on the CPs in mosquitoes [16,18,19]. RNAi-mediated knockdown of *DOPAL* synthase in *Ae. aegypti* larvae caused procuticle, exocuticle, and endocuticle thinning and changed the cuticular surface texture, which could lead to poor flexibility of the integument [15], and significantly delay the development of larvae, which, in turn, affected larval metamorphosis. In addition, changes in the expression of a wide range of genes encoding CPs, chitin, and genes involved in lipid biosynthesis were found during transcriptome analysis of the mosquitoes after *DOPAL synthase* knockdown [15]. In these data, two new ELO genes, *AeELO2* and *AeELO9*, were found to be involved in the development of larvae.

A recent study reported that acetyl-CoA carboxylase (ACC) and fatty acid synthase-1 (FAS1) are involved in lipid biosynthesis and the formation of eggshells after digestion of a blood meal in female *Ae. aegypti.* Their functions are different; mosquitoes deficient in ACC, but not FAS1, produced defective eggshells, and mosquitoes deficient in FAS1 had delayed blood meal digestion [20]. ACC is the first rate-limiting enzyme during fatty acid elongation and FAS is the multifunctional fatty acid synthase in eukaryotes (Figure 1). The research considered that decreased malonyl-CoA levels specifically inhibit fatty acid elongase function in mosquitoes [20]. In mammals, ELOs also have an important role in the biosynthesis of fatty acids possessing ≥ 18-carbon atoms [4,21]. However, little is known about the expression and biological function of ELOs in mosquitoes. In our study, we found that the expression of lipid biosynthesis genes was also significantly affected by RNAi-mediated knockdown of *DOPAL* synthase in mosquitoes [15]; among these lipid biosynthesis genes, the differential expressions of *AeELO2* and *AeELO9* are significant. Therefore, the functions of *AeELO2* and *AeELO9* were investigated using the RNAi in *Ae. aegypti* larvae and adults. Our data suggest that *AeELO2* and *AeELO9* play a vital role in regulating the mosquito development by functioning on the cuticular structure formation in larvae and fecundity in the adults of *Ae. aegypti*.

## 2. Materials and Methods

### 2.1. Mosquito Rearing

*Ae. aegypti* mosquitoes (the Rockefeller strain, provided by the Beijing Institute of Microbiology and Epidemiology, Beijing, China) were used in this study. Mosquitoes were reared as described previously [22]. 

### 2.2. Identification of AeELO2 and AeELO9

The identification of *AeELO2* and *AeELO9* was performed using bioinformatic approaches. The other known insect (*Drosophila melanogaster*; *Nilaparvata lugens*; *Homo sapiens*; *Tenebrio molitor*; *Saccharomyces cerevisiae*) ELO proteins were selected from published sources [6,8,11,23,24,25] and the corresponding protein sequences were obtained from the NCBI database. Multiple sequence alignment analysis was performed using MEGA6. The conserved histidine motif HXXHH (solid line) and the conserved motif YXYY (dotted line) are indicated.

### 2.3. Quantitative Real Time PCR

Spatiotemporal expression patterns of *AeELO2* and *AeELO9* were established by quantitative real time PCR (qPCR). *Ae. aegypti* eggs at different developmental stages were collected every 12 h from the beginning to the fifth day of development, and the total RNA was extracted. Then, larvae from different developmental stages, pupae, and female and male adult mosquitoes were collected. Different body parts of adults were dissected, and total RNA was extracted. The relative expression levels of *AeELO2* (XM_021847806.1) and *AeELO9* (XM_021837993.1) at different developmental stages and in different body parts of adults were analyzed. The following qPCR primers were used to detect *AeELO2* (forward: 5′-TTG ACC ATC ATT GCC AG-3′ and reverse: 5′-CCA TCC AAG CCC TCG TA-3′) and *AeELO9* (forward: 5′-CTC ACA GAC ATA CAC GA-3′ and reverse: 5′-TAA AGT AGG CAT AGA AT-3′). *RSP17* was used as an internal reference gene [26].

### 2.4. RNA Interference in Larvae and Adult Mosquitoes

Total RNA was extracted at the developmental stage with the highest relative expression of *AeELO2* and *AeELO9*, and cDNA was synthesized with the PrimeScript™ RT reagent kit (TaKaRa, Dalian, China). The primers (forward: 5′-ACT GTT CTA CGA GGG CTT G-3′ and reverse: 5′-CAT TTG GCA GAG TGA-3′) were used to amplify a fragment of *AeELO2* and the primers (forward: 5′-AGG AAC CTA CTC GTG GGC TA-3′ and reverse: 5′-AAC TGG TGC GTG AAG A-3′) were used to amplify a fragment of *AeELO9*. After amplification, the amplified fragment was ligated into the pL4440 vector used for interference and transformed into HT115 (DE3) competent cells. Bacteria were cultured in LB medium until the optical density of the solution at 600 nm (OD_600_) was in the 0.4–0.6 range, followed by the induction by the addition of isopropyl β-D-1-thiogalactopyranoside (IPTG) for 4–6 h. Bacteria were collected and harvested 4–6 h after induction, and the dsRNAs were extracted from the collected bacteria. Mosquito larvae were fed with chitosan-coated dsRNA nanoparticles, and the adult mosquitoes were microinjected with dsRNA [15].

### 2.5. Cold Treatment of Mosquito Larvae and Adults

The third or fourth instar larvae of the control groups and *AeELO2* and *AeELO9* knockdown groups were collected. A total of 20 larvae were collected from each group. Each larva was separately placed into a 1.5 mL centrifuge tube containing 500 µL sterile water. Then, these tubes were placed at 4 °C, and the larval mortality was determined every 12 h. The experiment was repeated three times. For adult mosquitoes, a total of 20 dsRNA injected adult mosquitoes were collected and placed at −20 °C for 3–4 min. Afterwards, the mosquito was placed at room temperature for 30 min and stimulated with a needle. If it did not move, it was regarded as dead. The experiment was repeated three times.

### 2.6. Statistical analyses

A one-way ANOVA test was performed to evaluate the significance of the differences between the groups. The threshold level for significance was *p* < 0.05.

## 3. Results

### 3.1. Identification of AeELO2 and AeELO9

In the *Ae. aegypti* ELO family, *AeELO2* and *AeELO9* were named after the third digits of their aliases *AAEL002673* and *AAEL009574*. Analyses of the predicted amino acid sequences in the NCBI search indicated that AeELO2 and AeELO9 indeed encoded ELOs as they shared characteristic domains of eukaryotic ELOs, including a HXXHH motif and a YXYY motif in the domain (Figure S1) [5].

### 3.2. Spatiotemporal Expression Profiles of AeELO2 and AeELO9 

**Figure 2:**
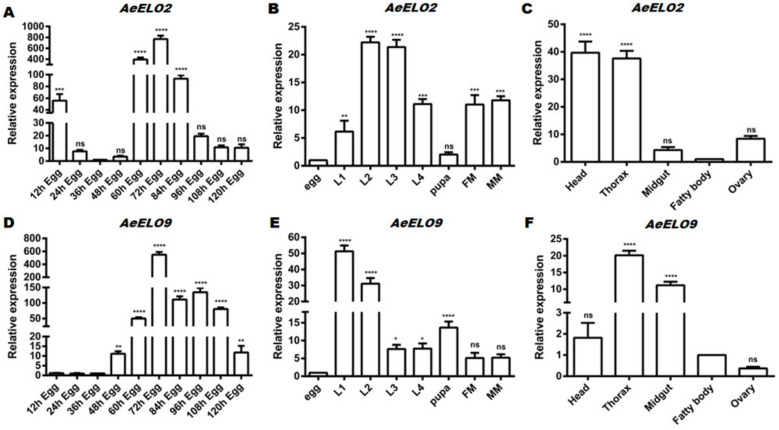
Expression levels of *AeELO2* and *AeELO9*. (**A**) Temporal expression levels of *AeELO2* at different times of egg development (12 h, 24 h, etc., represent the time after egg deposition). Eggs at 36 h were used as the control. (**B**) Temporal expression levels of *AeELO2* at different developmental stages. Eggs at 36 h were used as the control. (**C**) *AeELO2* expression in different body parts of adults. The fatty body was used as the control. (**D**) Temporal expression levels of *AeELO9* at different times of egg development (12 h, 24 h, etc., represent the time after spawning). Eggs at 36 h were used as the control. (**E**) Temporal expression levels of *AeELO9* in different developmental stages. Eggs at 36 h were used as the control. (**F**) *AeELO9* expression in different body parts of adults. The fatty body was used as the control. *Abbreviations*: L1, first instar larvae; L2, second instar larvae; L3, third instar larvae; L4, fourth instar larvae; FM, female adults; MM, male adults. * *p* < 0.05, ** *p* < 0.01, *** *p* < 0.001, **** *p* < 0.0001, ns, not significant. *, **, etc., represent a statistically significant difference compared with the control.

Analysis of the RNA-seq database following RNAi-mediated knockdown of *DOPAL* synthase in *Ae. aegypti* showed that *AeELO2* expression was up-regulated, while that of *AeELO9* was down-regulated (Figure S2A–C). At different stages of egg development, the relative expression level (the expression level was set to 1 at 36 h after oviposition) of *AeELO2* was the highest at 60–84 h after oviposition, and peaked at 72 h after oviposition (Figure 2A). The egg stage of *Ae. aegypti* was used as the control for our studies of the complete life cycle. *AeELO2* expression was the lowest at the egg and pupal stages, the highest in 2nd and 3rd instar larvae, and intermediate for the adult mosquitoes (Figure 2B). *AeELO2* was predominantly expressed in the head and thorax, with relatively low expression in the ovary, fatty body, and midgut (Figure 2C). Similar to *AeELO2*, the relative expression of *AeELO9* peaked in eggs 72 h after egg laying (Figure 2E). However, *AeELO9* expression was relatively high at the late stage of egg development (from 48 h to 120 h after egg laying) (Figure 2D). The results showed that the highest *AeELO9* expression was in the 1st and 2nd instar larvae, followed by the pupa stage (Figure 2E). Unlike *AeELO2*, *AeELO9* had the highest expression level in the thorax and midgut of female adult mosquitoes, but not in the head (Figure 2F). Like *AeELO2*, the expression of *AeELO9* in the ovary and fatty body was relatively low (Figure 2F).

### 3.3. RNAi-Mediated Knockdown of AeELO2 Affects Larval Molting Behavior

**Figure 3:**
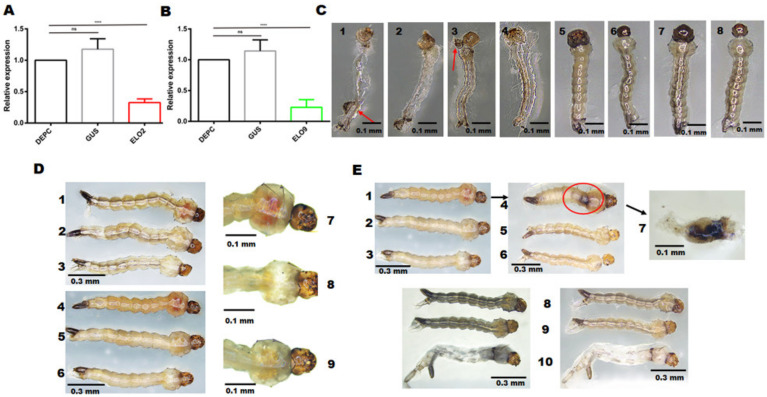
Changes in larval phenotype after RNAi-mediated *AeELO2* knockdown. (**A**) Quantitation of *AeELO2* expression in larvae after RNAi-mediated knockdown. (**B**) Quantitation of *AeELO9* expression in larvae after RNAi-mediated knockdown. (**C**) Abnormal molting caused by *AeELO2* knockdown. Here, 1, 2, 3, and 4 represent abnormally molting larvae in the *AeELO2* knockdown group; 5 and 6 are the larvae in the *gus*-dsRNA treatment group; 7 and 8 represent the blank control group. (**D**) *AeELO2* knockdown results in a red thorax. Here, 1 and 4 are the abnormal larvae after RNAi-mediated *AeELO2* knockdown; 2 and 5 represent the larvae in the negative control group (*gus*-dsRNA treated); 3 and 6 are the larvae in the blank control group (water treated). (**E**) The larvae with a red thorax became swollen for a short time after death. Here, 1 represents the larvae with a red thorax in the *AeELO2* knockdown group; 2 and 3 represent the larvae in the control groups; 4 represents the larvae within 12 h after death; 5 and 6 represent the larvae in the control groups at 12 h after death; 7 shows the dissected state of the larva shown in 4. In addition, 8 and 9 show larvae from the control groups; 10 represents larvae from the *AeELO2* knockdown group. *Abbreviations*: DEPC, blank control, larvae treated with diethyl pyrocarbonate water; GUS, larvae treated with *gus*-dsRNA; ELO2, larvae treated with *AeELO2*-dsRNA; ELO9, larvae treated with *AeELO9*-dsRNA; **** *p* < 0.0001, ns, not significant.

The quantitative results indicated that *AeELO2* and *AeELO9* mRNA levels are significantly decreased with RNAi, by 75% ± 2.3% (*p* < 0.0001) and 70% ± 4.5% (*p* < 0.0001), respectively, compared with the control groups (Figure 3A,B). Moreover, 32% ± 3.3% of the 2nd and 4th instar larvae died due to abnormal molting after *AeELO2* knockdown. Specifically, the 2nd instar larvae died during ecdysis (Figure 3C). A part of the molted cuticle of the dead larvae remained in the trunk of the body (as indicated by the red arrow, showing that the old head cuticles remain on the surface of the new larva). In addition, the body contents were concentrated at the junction of the abdomen and the thorax in some abnormally molting 4th instar larvae, while both old and new cuticles were stacked at the end of the abdomen (Figure 3D). In addition, 40% ± 3.5% of the *AeELO2-*deficient 4th instar larvae appeared in red color on the thorax (Figure 3D). The body of larvae with a red thorax appeared swollen, and the junction between the thorax and abdomen became obviously black within 12 h after death (Figure 3E). The anatomical examination revealed that the blackened part was not the larval cuticle, but internal tissue fluid, which could be transferred to the midgut (Figure 3E). However, unlike *AeELO2*, *AeELO9*-deficient larvae did not show abnormal molting (Figure S3).

### 3.4. The Effects of AeELO2 and AeELO9 on Larval Development and Freezing Tolerance

**Figure 4:**
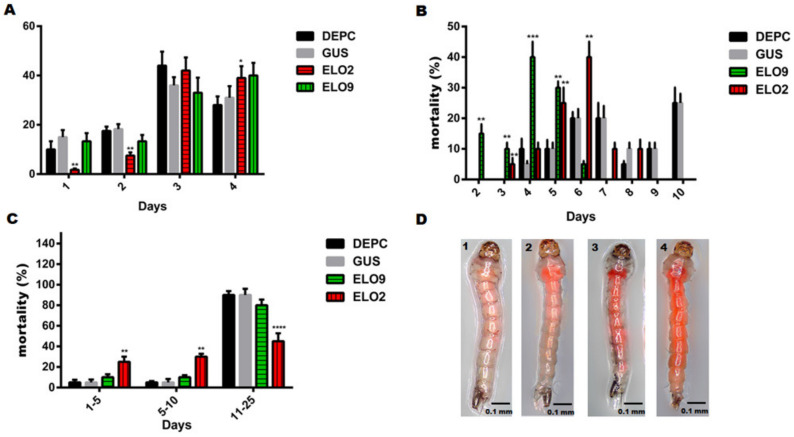
Effects of *AeELO2* and *AeELO9* knockdown on the growth rate and cold tolerance of larvae and pupae. (**A**) Larvae to pupae development rate. The days after treatment and the percentage of pupae are shown on the x- and y-axes, respectively. Each treatment group contained 120 larvae, and the experiment was repeated three times. (**B**) Mortality rate of the 4th instar larvae at 4 °C. The days after treatment and mortality rate (%) are shown on the x- and y-axes, respectively. (**C**) Mortality rate of pupae at 4 °C. The days after treatment and mortality rate (%) are shown on the x- and y-axes, respectively. (**D**) The osmotic balance of larval cuticle. The larvae of different treatment groups were soaked with Eosin Y dye for 5 min. Here, 1 represents the larvae of blank control; 2 represents the larvae treated with *gus*-dsRNA; 3 represents the larvae in the *AeELO2*-dsRNA treatment group; 4 represents the larvae treated with *AeELO2*-dsRNA. Each treatment group contained 30 larvae, and the experiment was repeated three times. *Abbreviations*: DEPC, blank control, larvae treated with diethyl pyrocarbonate water; GUS, larvae treated with *gus*-dsRNA; ELO2, larvae treated with *AeELO2*-dsRNA; ELO9, larvae treated with *AeELO9*-dsRNA; * *p* < 0.05, ** *p* < 0.01, *** *p* < 0.001, **** *p* < 0.0001, ns, not significant. *, **, etc., represent a significant difference compared with the blank control (DEPC water-treated).

Taking the day when the larvae begin to pupate as the first day, the pupation rates of larvae in the control groups were 17% ± 2.5% on the first and second day (Figure 4A). The pupation rate in the *AeELO9* knockdown group was similar to that in the control groups (Figure 4A). However, the pupation rate of *AeELO2* knockdown larvae was significantly lower than that of the control larvae, at 5% ± 1.3% on the first and second days (Figure 4A). *AeELO2*-knockdown larvae pupation was delayed 1-2 days compared with the control larvae (Figure 4A). Therefore, *AeELO2* knockdown significantly inhibited the development from larvae to pupae, whereas *AeELO9* knockdown larvae were not affected.

Next, survival at 4 °C was determined for larvae (3rd–4th instar) and pupae in the *AeELO2* and *AeELO9* knockdown and control groups. The mortality rate of *AeELO9* knockdown larvae was 15% ± 4.2% on the second day at 4 °C, and the mortality rate peaked on the fourth day. All the larvae died on the sixth day (Figure 4B). Unlike *AeELO9* knockdown, *AeELO2* knockdown larvae and pupae started dying after 3 days at 4 °C. Their mortality rate gradually increased in the following days, with the highest mortality observed on the sixth day. Compared with *AeELO9* knockdown group, all *AeELO2* knockdown larvae died on the tenth day (Figure 4B). In addition, pupae were also collected and kept at 4 °C. A total of 90% ± 2.3% of the control and *AeELO9* knockdown pupae were still alive after 11 days at 4 °C, while only 45% ± 5.3% of *AeELO2* knockdown pupae survived after 11 days (Figure 4C).

The 4th instar larvae of different treatment groups were soaked with Eosin Y dye for 5 min and observed under a microscope after washing with deionized water (five times). The larvae of the *AeELO2*-dsRNA and *AeELO9*-dsRNA treated groups contained more of the Eosin Y dye, relative to the control larvae. This indicated that the osmotic balance of *AeELO2*-dsRNA and *AeELO9*-dsRNA treated larvae (52% ± 3.3%) was destroyed (Figure 4D). 

### 3.5. Decreased AeELO2 and AeELO9 Expression Inhibited Egg Hatching

**Figure 5:**
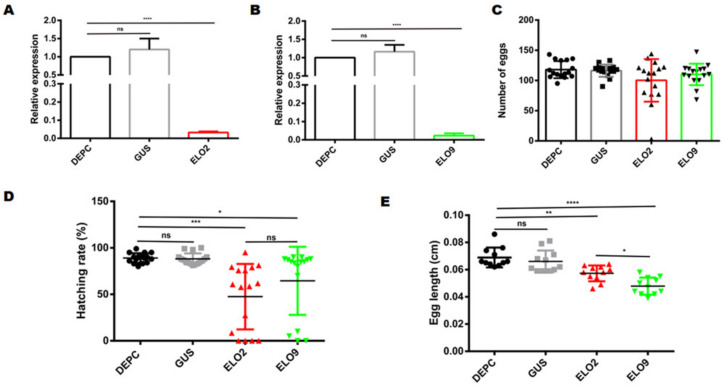
Effects of RNAi-mediated knockdown of *AeELO2* and *AeELO9* on egg production and hatchability. (**A**) RNAi-mediated knockdown of *AeELO2*. (**B**) RNAi-mediated knockdown of *AeELO9*. (**C**) Changes of oviposition in adult mosquitoes after injection with dsRNAs targeting *AeELO2* and *AeELO9*. The statistical unit is a single mosquito. (**D**) Effect of RNAi-mediated knockdown of *AeELO2* and *AeELO9* on egg hatching rates. (**E**) RNAi-mediated knockdown of *AeELO2* and *AeELO9* reduced egg length. *Abbreviations*: DEPC, blank control, adult mosquitoes treated with diethyl pyrocarbonate water; GUS, adult mosquitoes treated with *gus*-dsRNA; ELO2, adult mosquitoes treated with *AeELO2*-dsRNA; ELO9, adult mosquitoes treated with *AeELO9*-dsRNA; * *p* < 0.05, ** *p* < 0.01, *** *p* < 0.001, **** *p* < 0.0001, ns, not significant. *, **, etc., represent a significant difference compared with the blank control (adult mosquitoes treated with DEPC water). Red represents *AeELO2* and green represents *AeELO9*; The black dot, gray box, red triangle, and green inverted triangle represent the number of eggs laid by DEPC water, gus-dsRNA, *AeELO2*, and *AeELO9*, respectively.

Quantitative PCR analysis showed that RNAi-mediated knockdown *AeELO2* and *AeELO9* in adult mosquitoes reduced expression level to 90% ± 3.3%, when compared with the control (Figure 5A,B). dsRNAs targeting *AeELO2* and *AeELO9* were injected into adult mosquitoes 12 h before blood feeding, and eggs were laid three days after a blood meal. There was no significant difference in the number of eggs laid between the control groups (adult mosquitoes injected with DEPC water and *gus*-dsRNA) and the treatment groups (adult mosquitoes injected with dsRNAs targeting *AeELO2* and *AeELO9*) (Figure 5C). However, the hatching rate of eggs in the *AeELO2* and *AeELO9* knockdown groups was significantly reduced. The hatching rate of *AeELO9* knockdown mosquitoes was 65% ± 2.3%, and that of *AeELO2* knockdown mosquitoes was only 47, while that of the control mosquitoes was more than 90% ± 5.3% (Figure 5D). Compared with the control groups, *AeELO2* and *AeELO9* knockdown significantly decreased the egg hatching rate, and the effect of *AeELO2* knockdown was greater than that of *AeELO9* knockdown. 

As shown in Figure 5D, the eggs laid by *AeELO2* and *AeELO9* knockdown mosquitoes were significantly shorter than those laid by control mosquitoes. The average egg length for the control mosquitoes was 0.67 ± 0.13 mm, while egg lengths were 0.57 ± 0.15 mm and 0.48 ± 0.11 mm in the *AeELO2* and *AeELO9* knockdown groups, respectively (Figure 5E). Therefore, *AeELO2* and *AeELO9* deficiency significantly reduced egg length compared with the controls, and *AeELO9* knockdown had a greater effect on egg length than the *AeELO2* knockdown (Figure 5E).

### 3.6. Inhibiting the Expression of AeELO2 and AeELO9 Interfered with Cuticle Elasticity and Osmotic Pressure during Adult and Egg Development

RNAi results suggested that *AeELO2* and *AeELO9* may not affect the number of eggs laid by a mosquito (Figure 5C), but 30% ± 3.3% of *AeELO2* and *AeELO9* knockdown adult mosquitoes had less oviposition (Figure S4A). Moreover, the mean egg production per *AeELO2* and *AeELO9* knockdown mosquito was 65, while that of the control mosquito was 110 ± 10 (Figure S4B). In addition, these adult mosquitoes exhibiting abnormal egg laying died three to five days after spawning, but control mosquitoes survived for ten days. 

**Figure 6:**
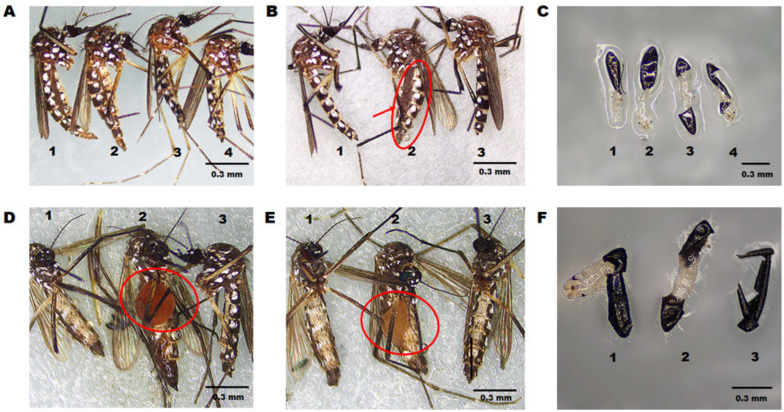
Effects of RNAi-mediated knockdown of *AeELO2* and *AeELO9* on adult mosquito cuticles and egg development. (**A**) Alterations in cuticular elasticity in adult mosquitoes after *AeELO2* knockdown. Here, 1, 2 show the *AeELO2* knockdown group; 3 and 4 show the *gus*-dsRNA-treated and DEPC water-treated control groups, respectively. (**B**) Same as panel a, and 1 and 3 represent the *gus*-dsRNA-treated and DEPC-water treated control groups, respectively; 2 represents the *AeELO2* knockdown group. (**C**) The phenotype of the hatched eggs, where 1 and 2 represent the *gus*-dsRNA-treated and DEPC water-treated control groups, respectively; 3 and 4 showed the *AeELO2* knockdown group. (**D**,**E**) Alterations in cuticular osmotic pressure in adult mosquitoes after *AeELO9* knockdown. Here, 1 and 3 represent the *gus*-dsRNA treated and DEPC water-treated control groups, respectively, in panels d and e; 2 *AeELO9-*dsRNA-treated mosquitoes. (**F**) Status of unhatched eggs in the *AeELO9* knockdown group. Here, 1 and 2 show the *gus*-dsRNA treated and DEPC water-treated control groups, respectively; 3 represents the *AeELO9* knockdown group. *Abbreviations*: DEPC, diethyl pyrocarbonate. (**A**,**B**,**D**,**E**) Each treatment group contained 30 adults, and the experiment was repeated three times. (**C**,**F**) Each treatment group dissected 60 unhatched eggs, and the experiment was repeated three times.

Microscopic observation showed that the abdomen of the control mosquitoes returned to its original state after oviposition and could support normal feeding behaviors. In contrast, the abdomens of *AeELO2* knockdown adult mosquitoes with abnormal oviposition remained in the egg-laying state with limited extension and did not return to the normal state (Figure 6A). In addition to the destruction of cuticle elasticity, 25% ± 5.2% of *AeELO2* knockdown adult mosquitoes died with unproduced eggs in their ovaries (Figure 6B). Whereas, *AeELO9* knockdown mosquitoes did not retain unproduced eggs, nor did they die during egg laying. However, 50% ± 4.5% of *AeELO9* knockdown mosquitoes died three days after oviposition with abdomens full of tissue fluid (red circle) (Figure 6D–E).

*AeELO2* deficiency resulted in reduced egg length (Figure 4E), but no other changes in egg appearance and structure were observed. After dissection, the mature 1st instar larvae could be observed (Figure 6C). Although no obvious abnormalities were observed in the egg phenotype of *AeELO9* knockdown mosquitoes, mature 1st instar larvae were not found in the eggs after dissection. As shown in Figure 6F, compared with the eggs of control mosquitoes, larvae did not develop in the eggs of *AeELO9* knockdown mosquitoes.

## 4. Discussion

Previous studies have shown that ELOs have critical roles in the insect’s survival. For example, RNAi-mediated knockdown of CG6660 (a gene encoding a predicted elongase) in *Drosophila* induced a lethal phenotype [6]. In *T. molitor*, silencing of *TmELO1* via RNAi resulted in an increased mortality rate [8], and *LmELO7* deficiency caused lethal phenotypes by decreasing cuticular hydrocarbon (CHC) amounts during *locust* molting [27]. In this study, two novel fatty acid elongase genes (*AeEL02* and *AeELO9*) were identified in *Ae. aegypti*. The results indicated that *AeELO2* and *AeELO9* are essential for the larva and adult survival of *Ae. aegypti*. *AeELO2* deficiency caused abnormalities in larval molting. Moreover, *AeELO2* and *AeELO9* deficiency led to the rapid death of adult mosquitoes after oviposition. *AeELO2* knockdown adult mosquitoes died because their abdominal cuticles were in an extremely extended state during the oviposition process. These results suggest that cuticle flexibility was affected. The innermost procuticle of insects contains the protein–chitin matrix, which is necessary for its stability. *DOPAL* synthase is involved in cuticular proteins cross-linking, which affects cuticular flexibility [15,16]. Following RNAi-mediated knockdown, the transcription of *AeELO2* decreased significantly. These results suggest that *AeELO2* may participate in the formation of flexible cuticles.

In *AeELO9*-deficient adult mosquitoes, the flexibility of the abdominal cuticles did not appear to be affected, but abdominal swelling was observed. Some studies have reported that insect CHCs are deposited in the outermost cuticle layer, forming a permeable barrier, which can effectively protect insects from the external environment. It can not only prevent water evaporation from the insect body, but effectively blocks external water, pathogens, chemical pesticides, and other harmful substances as well [3,28]. *LmELO7* is involved in CHC synthesis in *L. migratoria*, and *LmELO7* knockdown affected the water retention and epidermal permeability of locusts [27]. It has been demonstrated in migratory locusts that inhibition of epidermal hydrocarbon synthesis can increase permeability to water-soluble substances [1,29]. Thus, it can be inferred that decreased *AeELO9* expression may disrupt CHC deposition in the outermost cuticles of insects. Disruptions in cuticle permeability can lead to abnormalities in water balance, abdominal swelling, and death.

Mutations of genes that affect lipid or carbohydrate metabolism can impact ovarian development, fertility, or embryogenesis [30,31]. In egg-laying animals, such as birds [32], reptiles [33], and insects, the developing embryo can only obtain nutrient input into the egg before oviposition. It is well established that lipids are the main energy source in eggs. In this report, we present data demonstrating that *AeELO2* and *AeELO9* are also involved in regulating egg size and hatching in mosquitoes. *AeELO2* and *AeELO9* knockdown significantly reduced the egg hatching rate. The mature 1st instar larvae could not hatch from *AeELO2*-deficient eggs. This may be due to damage to the cuticular structure. This was also evident from the abnormal molting development and growth of *AeELO2*-deficient larvae. Further, the effect of *AeELO2* knockdown on egg size could also be due to any disruption of lipid synthesis or impact on cuticle elasticity, which could lead to the squeezing of eggs during oviposition, resulting in shorter egg length.

In addition, no larvae were found in the unhatched eggs from *AeELO9* knockdown mosquitoes. Early research showed that in the process of egg development, especially during the second half, the lipid transfer from egg yolk to embryo increases [34]. Considering that *AeELO9* is expressed mainly during the late stage of egg development, it is possible that *AeELO9* may affect the biosynthesis of lipids from the blood meal, thereby affecting lipid transfer and egg development.

In general, the biological function of ELOs is not conserved in complete metamorphosis and incomplete metamorphosis insects. *AeELO2* and *AeELO9* affect the development of larvae and the flexibility or osmotic balance of adult cuticles, which not only greatly reduces the reproductive ability of *Ae. aegypti*, but also makes them a potential target for future pesticides.

## 5. Conclusions

*AeELO2* and *AeELO9* play crucial roles in larval development and in the adult fecundity of *Ae. aegypti*. *AeELO2* mainly participates in larval molting and growth, and regulates the flexibility and elasticity of adult mosquito cuticles. *AeELO9* affects the cold resistance of larvae and adult mosquitoes, and also regulates the permeability of adult mosquito cuticles and egg development, thereby affecting the reproduction of *Ae. aegypti*.

## Data Availability

The data used to support the findings of this study are included in the article.

## Supplementary Materials

The following supporting information can be downloaded at https://www.mdpi.com/article/10.3390/insects14020189/s1, Figure S1: Amino acid alignment of AeELO2, AeELO9, and other known insect ELO proteins; Figure S2: Quantitative RCR was used to determine the relative expression levels of *AeELO2* and *AeELO9* after *DOPAL* synthase knockdown; Figure S3: Mortality of larvae molted abnormally; Figure S4: The probability of reduced oviposition and the number of eggs in adult mosquitoes after *AeELO2* and *AeELO9* knockdown.
