# Supplementary material for: Elongases of Long-Chain Fatty Acids ELO2 and ELO9 Are Involved in Cuticle Formation and Function in Fecundity in the Yellow Fever Mosquito, Aedes aegypti
Source: Insects. 2023 Feb 14;14(2):189. doi: 10.3390/insects14020189 (PMC9961117; doi:10.3390/insects14020189)
Supplement: Supplementary file 1 [file insects-14-00189-s001.zip › supplementary.pdf]

Supporting information

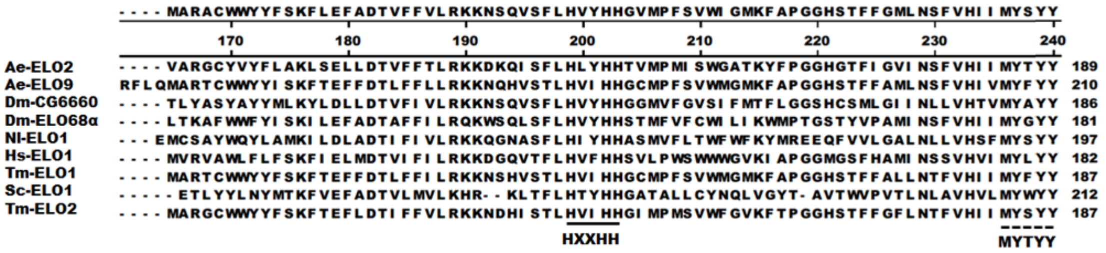

Figure S1. Amino acid alignment of AeELO2, AeELO9 and other known ELO proteins. (Ae, *Aedes aegypti*; Dm, *Drosophila melanogaster*; Nl, *Nilaparvata lugens*; Hs, *Homo sapiens*; Tm, *Tenebrio molitor*; Sc, *Saccharomyces cerevisiae*). The other known ELO proteins were selected from published sources. The conserved histidine motif HXXHH (solid line) and the conserved motif YXYY (dotted line) are indicated.

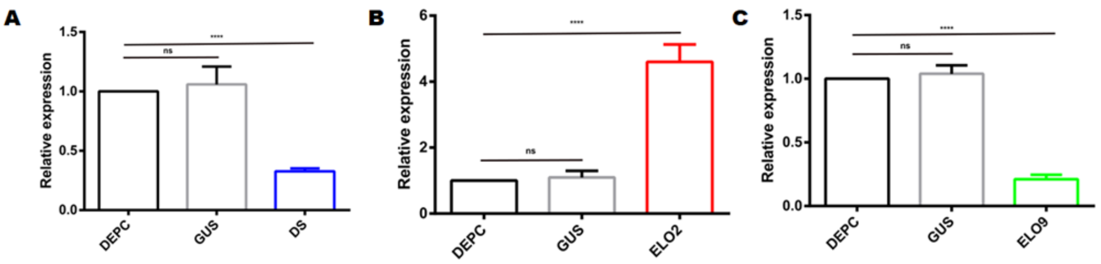

Figure S2. Quantitative RCR was used to determine the relative expression levels of *AeELO2* and *AeELO9* after *DOPAL* synthase knockdown. (A) Quantitation of *DOPAL* synthase expression after RNAi-mediated knockdown in larvae. (B) The relative expression of *AeELO2* after *DOPAL* synthase knockdown. (C) The relative expression of *AeELO9* after *DOPAL* synthase knockdown. *Abbreviations*: DEPC, larvae treated with DEPC water; GUS, larvae treated with *gus*-dsRNA; DS, larvae treated with *DOPAL* synthase-dsRNA; ELO2, larvae treated with *AeELO2*-dsRNA; ELO9, larvae treated with *AeELO9*-dsRNA; **** $P < 0.0001$, ns, not significant. **** and ns represent significant differences compared with the blank control (larvae treated with DEPC water).

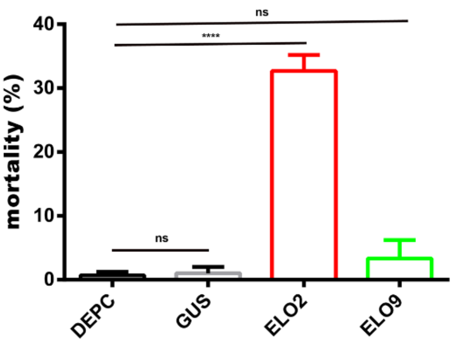

Figure S3. Mortality of larvae molted abnormally. *Abbreviations*: DEPC, Blank control; GUS, larvae treated with *gus*-dsRNA; ELO2, larvae treated with *AeELO2*-dsRNA; ELO9, larvae treated with *AeELO9*-dsRNA; **** $P < 0.0001$, ns, not significant. **** and ns represent significant differences compared with the blank control (larvae treated with DEPC water).

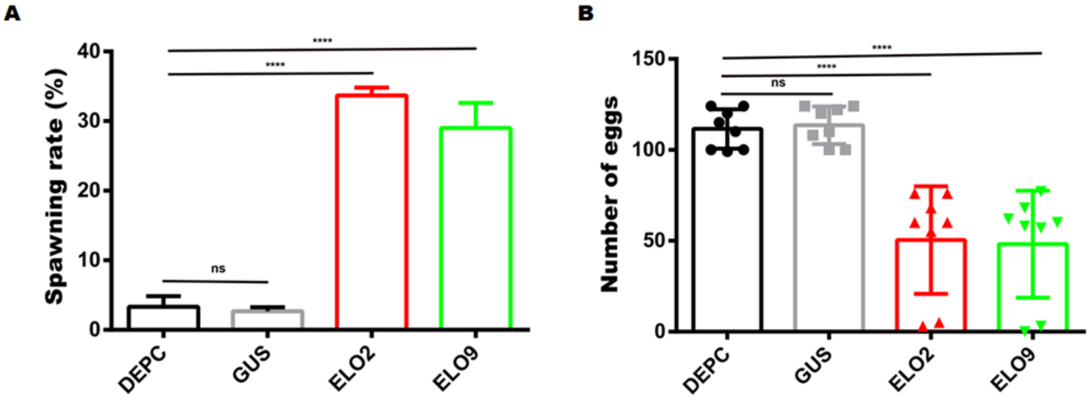

Figure S4. The probability of reduced oviposition and the number of eggs in adult mosquitoes after *AeELO2* and *AeELO9* knockdown. (A) The probability of oviposition in *AeELO2* and *AeELO9* knockdown mosquitoes was significantly lower than that in the controls. The different treatment groups and the probability of reduced oviposition are shown on the x- and y-axes, respectively. (B) The number of eggs laid by adult mosquitoes with reduced oviposition. *Abbreviations*: DEPC, adult mosquitoes treated with DEPC water; GUS, adult mosquitoes treated with *gus*-dsRNA; ELO2, adult mosquitoes treated with *AeELO2*-dsRNA; ELO9, adult mosquitoes treated with *AeELO9*-dsRNA; **** $P < 0.0001$, ns, not significant. **** and ns represent significant differences compared with the blank control (adult mosquitoes treated with DEPC water).
